# Prevalence of beta-lactam allergy: a retrospective chart review of drug allergy assessment in a predominantly pediatric population

**DOI:** 10.1186/s13223-016-0165-6

**Published:** 2016-11-29

**Authors:** Elissa M. Abrams, Andrew Wakeman, Tom V. Gerstner, Richard J. Warrington, Alexander G. Singer

**Affiliations:** 1 Section of Allergy and Immunology, Department of Pediatrics and Child Health, University of Manitoba, Winnipeg, Canada; 2 University College Dublin, Dublin, Ireland; 3 Section of Allergy and Immunology, Department of Internal Medicine, University of Manitoba, Winnipeg, Canada; 4 Department of Family Medicine, University of Manitoba, Winnipeg, Canada

**Keywords:** Anaphylaxis, Beta-lactam allergy, Drug allergy, adverse drug reaction

## Abstract

**Background:**

Research suggests that 90% of patients labeled beta-lactam allergic are able to tolerate penicillins following further assessment. This study aims to define and describe the frequency of true beta-lactam allergy following allergy patient evaluation in a predominantly pediatric population.

**Methods:**

306 primary care patients referred between January 2010 and June 2015 were assessed for a suspected beta-lactam allergy. Patient demographics, history and test results were extracted from electronic medical records. Testing performed was based on specialist recommendation following review of patient history.

**Results:**

34% of the study participants had intradermal testing. Oral challenge was given to 96.7% of the sample. 96% of patients with a prior history of beta-lactam allergy were advised that they could re-introduce beta-lactam antibiotics following evaluation.

**Conclusions:**

Among patients with a documented beta-lactam allergy or a recent history of a reaction there is a low rate of ‘true’ beta-lactam allergy. Consistent evaluation of beta-lactam antibiotic allergies can reduce rates of broad spectrum antibiotic prescribing, among other harmful consequences.

## Background

Approximately 10–20% of the general population have been labeled as penicillin allergic [1–3]. However, studies have clearly documented that over 90% of patients labeled allergic are able to tolerate penicillins once assessed [2, 4]. In addition, recent estimates suggest that the rate of penicillin allergy may be decreasing [5], particularly in pediatric populations [6]. This may be due to the changing nature of antibiotic prescription patterns. The reduction in intravenous penicillin use and the increase in oral amoxicillin usage may have reduced true allergies while increasing likelihood of viral rashes being inappropriately defined as “allergies” [6]. In addition, up to 80% of true IgE allergic patients will lose their sensitivity over time [7, 8], further contributing to reductions in penicillin allergy rates. For example, in a study of 740 patients with a history of beta-lactam antibiotic reactions, prevalence of positive skin testing was related to time elapsed since clinical reaction, in cases that reacted, 93% had a positive skin test in the past year, while this rate dropped to 22% in those who were evaluated 10 years or more after a reaction [8].

In 2015, the American Academy of Allergy Asthma and Immunology (AAAAI) released a statement requesting urgent action on the specified harms of erroneously labeling patients with penicillin allergy [9]. In particular, patients reporting penicillin allergies are more likely to receive broad-spectrum antibiotics [10] which have been linked to higher rates of antibiotic resistance [11] and higher health care costs in the community [12] and hospital [13]. Furthermore, while the causation remains to be elucidated, penicillin allergies recorded in hospital Electronic Health Records correlate strongly with longer stays and more iatrogenic infections [14]. While aiming to protect patients from adverse drug reactions, inappropriately using conservative documentation of penicillin allergy confers direct patient harm and is an unnecessary cost to the healthcare system [9–14].

This study aims to define and describe the frequency of true penicillin allergy among primary care patients evaluated by Allergy and Immunology specialists. It attempts to determine whether penicillin allergy could be removed from patient charts, to avoid the negative consequences of that labelling.

## Methods

### Study design

This retrospective chart review includes 306 predominantly pediatric patients referred from community primary care providers (family physicians or pediatricians) to assess a suspected allergy to beta-lactam antibiotics by two participating allergy and immunology specialists in Winnipeg, Manitoba, Canada. The outcome measures used were based on the recommendations made by the two participating allergy specialists, both fellows of the Royal College of Physicians of Canada. Consultations are publicly funded by Manitoba Health with no direct cost to the patient.

The inclusion criterion included all patients with a consultation regarding suspected beta-lactam allergy from January 2010 to June 2015. Patients were excluded if they had incomplete follow-up or were not evaluated for beta-lactam allergy in the clinic. In addition, intradermal testing and oral challenge were delayed at least 6 weeks after a clinical reaction.

Approval was obtained from the Human Research Ethics Board of the University of Manitoba. Individual participant consent was not obtained in accordance with Canada’s *Tri*-*Council Policy Statement: Ethical Conduct for Research Involving Humans* and the University of Manitoba Research Ethics board policy regarding retrospective chart reviews.

Patient data was extracted from electronic medical records (JonokeMed 5.5.3) and collated into a spreadsheet profiling patient age, gender, history and symptoms of adverse drug reactions, test results, and consultant recommendations. Queries captured patients with a visit to a participating physician between January 1, 2010 to July 2015. Data was collected in July 2015 by a single study author and validated by the consultants for accuracy. Patients were selected if they had either a visit coded with ICD9 code 995.20 for a drug allergy or a billing (fee) tariff corresponding to administration of an oral provocation challenge. Documentation from patient visits were reviewed for key terms “icil” or “amox” in the context of a reaction history or allergy test. All patient charts underwent a review for a referral and consultation regarding beta-lactam allergy. Relevant chart information was extracted from notes relating to initial and follow-up appointments.

The recommendations made by the consultants were primarily based on results from beta-lactam allergy testing, performed by protocols laid out by the American Academy of Allergy, Asthma and Immunology [2]. Those with recent adverse drug reaction (ADR) histories of non-urticarial rash and pruritus were orally challenged with appropriate dosing of amoxicillin or other implicated beta-lactam antibiotic [15]. Patients having remote or vague histories and those with histories more suggestive of a type I reaction received intradermal testing with later oral drug provocation challenge to the implicated beta-lactam antibiotic, if intradermal testing was negative. Intradermal testing was done with standard concentrations of penicilloyl polylysine (6 × 10^−5^ M), benzylpenicillin (10,000 U/mL) and ampicillin (1.25 mg/mL). Intradermal testing was read 15 min after administration. Delayed intradermal testing was not done, however patients were instructed to contact the clinic should any late reactions occur. Patients with histories suggestive of serious delayed-type reactions such as serum sickness or Stevens-Johnson were not tested and recommended to avoid beta-lactam antibiotics.

## Results

**Fig. 1:**
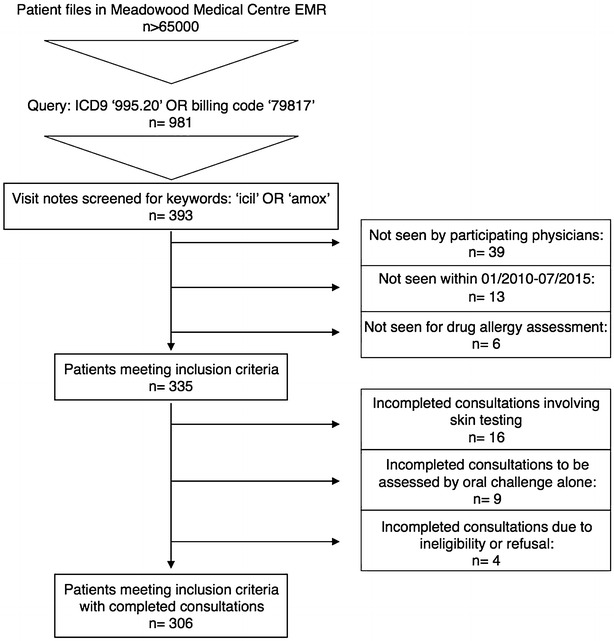
Inclusion criteria

There were 335 unique patient records meeting the study criteria. A total of 29 patients were excluded from the study due to pending test results (25 patients), refusal of oral challenge (3 patients) and a history of chronic urticaria with incomplete testing (1 patient). A total of 306 patients were considered as part of the study. Figure 1 displays the study inclusion criteria.

### Study population

303 (99%) of the patients were referred by their primary care providers (family physicians, pediatricians or nurse practitioner) the remainder were referred by an Emergency Department or other specialist providers. The sample was evenly distributed between the genders, with males being slightly younger on average at testing (Table 1). The mean age of the patient population was 11.6 years (standard deviation 17.3 years) and the median age was 6.0 years. Our sample was mostly pediatric patients although the large standard deviation reflects a small number of adult and older patients who skew the distribution to a bimodal pattern.

**Table 1:** Patient demographics

	Total	Male	Female
Number	306 (100%)	150 (49%)	156 (51%)
Age (Mean, SD)	11.6, 17.3	9.5, 16.3	13.6, 18.0
(Mode)	1	2	3
(Q1, Q2, Q3)	3, 6, 11	2, 5, 10	3, 7, 14
(Extremes)	0, 89	0, 89	0, 82

**Fig. 2:**
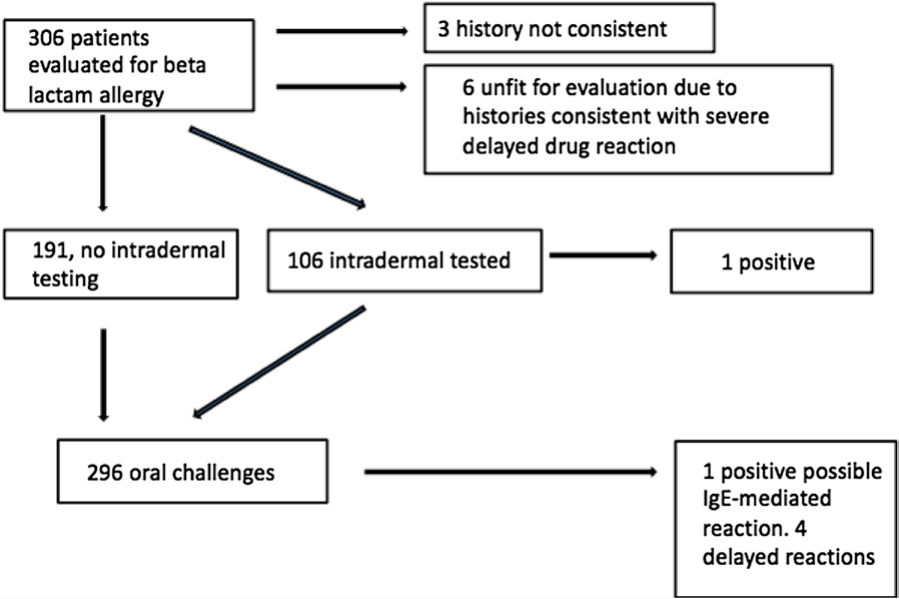
Patient evaluation

Patients had a variety of clinical reactions upon beta-lactam antibiotic exposure with the most prevalent “non-urticarial skin reactions”, “Urticarial skin reactions” and “multi-systemic complaints”. Table 2 outlines patient clinical reactions. Average time elapsed between drug reaction and allergological work up was 5.4 years. A total of 106 patients (34%) had intradermal testing (see Fig. 2). Oral challenge was given to 96.7% of the sample (296 patients), 64.5% (191 patients) of those patients had a history inconsistent with type 1 allergy and therefore did not receive prior skin testing. Type 1 allergy was found among 0.7% of the sample: 0.3% (1 patient) had a positive intradermal test and 0.3% (1 patient) with a negative intradermal test had a positive oral challenge potentially consistent with a type 1 reaction. This reaction (acute onset abdominal pain and emesis within an hour of a dose of amoxicillin) was different than the reaction that this patient initially presented with (acute urticaria within one hour after a dose of amoxicillin), and occurred in a toddler.

**Table 2:** Clinical reaction upon beta-lactam antibiotic exposure

Reaction	Number of patients
GI complaints	2
Unknown skin reaction (remote Hx)	18
Non-urticarial skin reactions	151
Urticarial skin reactions	76
Skin peeling noted	2
Multi-systemic complaints	50
Skin rash with fever/malaise	5
Describing open sores	1
Skin rash with GI complaints	6
Edema or difficulty breathing	34
Myalgia or joint swelling	5
History unknown	8

Additionally, 1.3% of the sample (4 patients) had a positive delayed oral challenge (delayed maculo-papular exanthems) after negative intradermal testing. In addition, 1.9% (6 patients) were advised to avoid beta-lactams without testing based on a clinical history suggestive of a serious delayed reaction (Table 3).

**Table 3:** Allergy type

Consensus allergy	Number	Percent (%)	CI_95_ (%)
Type 1	2	0.7	0–2.6
Type 2	0	–	–
Type 3 (serum sickness)	5	1.6	0.5–3.9
Type 4 (mild)	4	1.3	0.3–3.5
Type 4 (SJS)	1	0.3	0–2.1

After completed consultation, 2/306 patients (0.7%, CI_95_ 0–2.6%) were considered to be at increased risk of future IgE-mediated reactions (one due to positive intradermal testing and one due to a reaction with oral challenge). In addition, 10 patients (3.3%, CI_95_ 1.7–6.0%) were considered to be at increased risk of delayed reactions (4 of whom had delayed maculopapular exanthems with oral challenge, and 6 of whom had no testing due to history suggestive of a serious delayed reaction such as serum sickness). Overall, 294 (96.1%, CI_95_ 93.2–97.9%) of those evaluated were advised that they could safely use beta-lactam antibiotics in the future. It is unknown whether these patients have used beta-lactam antibiotics since their allergy evaluation.

## Discussion

This current study found that among patients with a label of beta-lactam allergy there was a low rate of true allergy observed. The overall rate of false positive reports of penicillin allergy was high with 96.1% of patients with a prior history of beta-lactam allergy advised that they could safely re-introduce beta-lactam antibiotics. The rate of true penicillin allergy in this sample was much lower than in other studies of referral populations, which have documented 10–20% true allergy among patients labeled as beta-lactam allergic [1–3]. Consistent evaluation of patients with a history of beta-lactam allergy could reduce the use of broad-spectrum antibiotics if performed before labelling patients with a presumed allergy. This practice could significantly reduce the risk of patients receiving broad spectrum antibiotics inappropriately, which is thought to contribute to antibiotic resistance. The Centers for Disease Control and Prevention report that in the United States there are about 2 million illnesses and 23,000 deaths caused by antibiotic-resistant bacteria [16]. The AAAAI has urged more aggressive use of drug allergy testing to reduce increasing rates of antibiotic resistance [9].

Previous studies have reported that 9–11% of patients have systemic reactions when skin testing is performed to beta-lactams [17, 18]. In a recent study 2.6% of beta-lactam allergic children reported mild to moderate reactions to skin testing [19]. Our study, in contrast, noted no systemic reactions to skin testing (although several possible explanations could exist for this finding).

In the current study, no patient with negative intradermal testing had a subsequent anaphylactic reaction on oral challenge. One patient had emesis and abdominal pain, which was considered to be potentially indicative of a type I reaction as it was shortly following oral challenge in a toddler. In addition, 1.3% (4 patients) had a delayed reaction on oral challenge, despite negative intradermal testing.

A possible limitation of our study was the lack of delayed intradermal readings and use of patch testing. For delayed beta-lactam reactions, some studies have suggested that either or both of these methods may provide some value [20–22]. In fact, some clinical management reviews recommend delayed intradermal testing and/or atopy patch testing in the evaluation of non-immediate reactions to penicillins [20]. Recent studies have also used 5-day drug provocation testing to evaluate non-immediate reactions to amoxicillin [23].

Our study followed the AAAAI practice parameter which recommends avoidance of testing and oral challenges in patients with a history of severe cutaneous adverse drug reaction such as serum sickness or Stevens Johnsons. Interestingly, other studies have noted a low correlation between reaction history for severe adverse drug reactions and subsequent risk of reactivity [24].

Low consensus between confirmed beta-lactam allergy and documented history of a beta-lactam allergy has implications for the implementation of a national agenda around interoperable digital patient health records among health care settings [25]. Interoperability is proposed to improve patient care and outcomes [25] but could present problems if erroneous clinical information is being shared due to the lack of patient record accuracy. Health care providers and administrators should be aware of the risk of sharing potentially erroneous patient data. These findings suggest the need to more actively incorporate strategies into primary care practices to proactively identify patients who are likely mislabeled with beta-lactam allergy to avoid the harms of antibiotic avoidance.

This study tracked consultation recommendations as oppose to more objective test results which was expected to produce a more reliable measure of the effects of penicillin allergy consultation in determining rates of valid avoidance of beta-lactams. Assessing the practices of only two physicians operating within one clinic can create the potential for bias related to clinical decision making. Our sample is primarily pediatric patients and it may not apply to other populations without assessment of local prevalence and demographics of ‘penicillin allergy’. Furthermore, this sample is dependent on the referral of primary care physicians so it is uncertain if certain patient populations with greater or lesser risk of mislabeling are being referred or not.

## Conclusion

Our findings demonstrate that a profound majority of pediatric patients in the community who had a consultation with an allergist erroneously consider themselves allergic prior to evaluation. Meanwhile, the risks of avoiding these targeted and often effective antibiotics have severe consequences for patients and the population. More research is needed to evaluate the prevalence of potential mislabeling of beta-lactam “allergy” and determine the true rate of type 1 reaction in the population.

Information regarding allergies that become part of patient records are primarily self-reported. As patients have increasing health literacy, more discussion will be required to clarify these labels. In addition, primary care providers need to be better informed as to when to refer patients for consultation with an allergist and how to properly record drug reactions. These types of initiatives are increasingly important as health information is shared across the system in systems that will eventually be interoperable.

This study highlights the widespread mislabeling of primary care patients with beta-lactam antibiotic allergy in a predominantly pediatric population and suggests urgent attention be paid to identifying these patients in order to determine who are truly allergic.

## Abbreviations

AAAAI: American Academy of Allergy Asthma and Immunology
ADR: adverse drug reaction
SJS: Stevens Johnson Syndrome
